# Dashboards to Support Implementation of the Quebec Alzheimer Plan: Evaluation Study With Regional and Professional Considerations

**DOI:** 10.2196/55064

**Published:** 2024-05-08

**Authors:** Genevieve Arsenault-Lapierre, Alexandra Lemay-Compagnat, Maxime Guillette, Yves Couturier, Victoria Massamba, Isabelle Dufour, Eric Maubert, Christine Fournier, Julie Denis, Caroline Morin, Isabelle Vedel

**Affiliations:** 1 Center for Research and Expertise in Social Gerontology Centre intégré universitaire de santé et de services sociaux du Centre-Ouest de l'Ile de Montréal Côte Saint-Luc, QC Canada; 2 Department of Family Medicine McGill University Montreal, QC Canada; 3 Lady Davis Institute for Medical Research Centre intégré universitaire de santé et de services sociaux du Centre-Ouest de l'Ile de Montréal Montreal, QC Canada; 4 Department of Social Work Sherbrooke University Sherbrooke, QC Canada; 5 National Insistute of Public Health of Quebec Quebec, QC Canada; 6 School of Nursing Sherbrooke University Sherbrooke, QC Canada; 7 Center on Aging Centre intégré universitaire de santé et de services sociaux de l’Estrie Centre hospitalier universitaire de Sherbrooke Sherbrooke, QC Canada; 8 Integrated University Health and Social Services Network of McGill University Montreal, QC Canada; 9 Integrated University Health and Social Services Network of Université de Montréal Montreal, QC Canada; 10 Integrated University Health and Social Services Network of Université Laval Quebec, QC Canada; 11 Integrated University Health and Social Services Network of Université de Sherbrooke Sherbrooke, QC Canada

**Keywords:** dashboard, learning health system, health policy, dementia care, health care regionalization, dementia, Alzheimer disease, qualitative, collaborative, focus group, primary care, implementation, attitude, opinion, perception, perspective, service, health care management

## Abstract

**Background:**

Health organizations face the critical task of executing and overseeing comprehensive health care. To address the challenges associated with this task, evidence-based dashboards have emerged as valuable tools. Since 2016, the regional health organizations of Quebec, Canada, have been responsible for ensuring implementation of the Quebec Alzheimer Plan (QAP), a provincial plan that aims to reinforce the capacity of primary care services to detect, diagnose, and treat persons with dementia. Despite the provincial scope of the QAP, the diverse material and human resources across regions introduce variability in the interest, utility, and specific needs associated with these dashboards.

**Objective:**

The aim of this study was to assess the interest and utility of dashboards to support the QAP implementation, as well as to determine the needs for improving these aspects according to the perspectives of various types of professionals involved across regions.

**Methods:**

An evaluative study using qualitative methods was conducted within a collaborative research approach involving different stakeholders, including the ministerial advisor and the four project managers responsible for supporting the implementation of the QAP, as well as researchers/scientific advisors. To support these organizations, we developed tailored, 2-page paper dashboards, detailing quantitative data on the prevalence of dementia, the use of health services by persons with dementia, and achievements and challenges of the QAP implementation in each organization’s jurisdiction. We then conducted 23 focus groups with the managers and leading clinicians involved in the implementation of the QAP of each regional health organization. Real-time notes were taken using a structured observation grid. Content analysis was conducted according to different regions (organizations with university mandates or nearby organizations, labeled “university/peripheral”; organizations for which only part of the territory is in rural areas, labeled “mixed”; and organizations in remote or isolated areas, labeled “remote/isolated”) and according to different types of participants (managers, leading clinicians, and other participants).

**Results:**

Participants from organizations in all regions expressed interest in these dashboards and found them useful in several ways. However, they highlighted the need for indicators on orphan patients and other health care providers. Differences between regions were observed, particularly in the interest in continuity of care in university/peripheral regions and the need for diagnostic tools adapted to the culture in remote/isolated regions.

**Conclusions:**

These dashboards support the implementation of an Alzheimer Plan and contribute to the emergence of a learning health care system culture. This project allows each region to increase its monitoring capacity for the implementation of the QAP and facilitates reflection among individuals locally carrying out the implementation. The perspectives expressed will guide the preparation of the next iteration of the dashboards.

## Introduction

Health organizations worldwide are encouraged to continually improve their performance by identifying areas in need of enhancement and implementing interventions to address them [1-3]. The learning health system approach posits that a system “learns” through a reflective or cyclical process that engages a community in an empirical analysis of data related to a problem, leading to the discovery of new knowledge and practices [1-4]. Dashboards are an important tool to support a learning health system as they allow tracking the performance of an organization or a specific process and identifying areas in need of improvement [5]. These visual and interactive tools are used to monitor, measure, control, and analyze the performance and outcomes of an organization or a specific process [6,7] and can assist decision-makers in making changes based on the obtained results [8,9]. Dashboards may include alerts, customization options, and contextual information [7,10].

Dashboards can serve decision-makers as well as health managers and professionals in focusing on the most critical activities, monitoring trends, analyzing and identifying areas in need of improvement, and making data-driven decisions [7]. They can foster reflection on the causes explaining results and adjusting actions to better achieve the intended goals [11]. In this sense, their use can be beneficial for improving the quality of care, reducing costs, and increasing efficiency in health care [12]. Additionally, dashboards reduce cognitive load, task completion time, and errors, and enhance situational awareness and adherence to evidence-based practice guidelines [12]. However, the diverse needs of different users (decision-makers, managers, or clinicians) can make it challenging to choose indicators in a dashboard that must be concise to fulfill its function [13,14]. These needs vary and depend on the clinical or managerial context, population characteristics, and the role of professionals [10], which can create information overload [6]. Indeed, health care managers in Canada often report the presence of too many indicators in dashboards, leading to confusion [13,15]. It is necessary to adapt the dashboards to professional contexts and consider the multiplicity of uses in their development and evaluation [16].

The use of a dashboard in national programs such as an Alzheimer Plan further increases the need for considering the multiplicity of uses, as these dashboards need to take into consideration regional differences, especially with respect to resources and populations. Dashboards have been used in the implementation of national programs for health improvement in many countries, including the United States, Canada, and Australia [17-19], for evaluating major neurocognitive disorders [20,21] or for improving care for these individuals [22]. To our knowledge, no study has reported the use of dashboards in the context of implementing an Alzheimer Plan.

Quebec is one of the first Canadian provinces to have developed and implemented an Alzheimer Plan [23]. The Quebec Alzheimer Plan (QAP; *Plan ministériel sur les troubles neurocognitifs majeurs*) gives interdisciplinary primary care clinic clinicians the responsibility to identify, assess, diagnose, treat, and follow individuals with dementia and their care partners [24,25]. The person-centered approach of the QAP is anchored in primary care, with specialized services supporting more complex clinical situations [24,25]. The QAP includes an implementation strategy supported by the development of professional and organizational capacities, the deployment of partnership governance, ambitious change management, and an independent evaluation by our research team [24,25]. For the generalization of the QAP to the entire province in 2016, the Ministry of Health and Social Services assigned the responsibility for implementing the QAP to the regional health organizations [23,25,26]. This involves training and mentoring doctors and health care professionals in each interdisciplinary primary care clinic in their territory and supporting implementation of the QAP. Four QAP project managers, one for each integrated university health and social services network overseeing regional health organizations [27], served as an interface between the Ministry of Health and Social Services and the health organizations in their territory. These four project managers support organizations to promote change and the sharing of experiences among organizations in their territory.

Health care needs and resources vary significantly from one organization to another based on regional factors such as geographic distribution and demographic characteristics of the population [1,28-30], as well as the availability of local health and social services [1,29,31,32]. For example, the higher population density [1] in urban regions can increase both health and social services needs and resources [1,32]. Although these regions benefit from more resources, access to emergency services, psychosocial services, services for older adults, and mental health services remains suboptimal in almost all regions [29]. Health organizations in more remote areas generally cover very large territories [33]. These organizations face challenges such as geographic accessibility issues [31,34], a lack of local resources across the care continuum [35-37], and an aging population with complex health needs [28,38]. It is important to consider these regional differences in the development of dashboards supporting the implementation of a large-scale Alzheimer Plan [39,40]. Thus, in 2020, the ministry entrusted our research team with the development of dashboards to support regional health organizations in their implementation activities. The objective of this study was to evaluate the interest and utility of these dashboards and to assess the needs for improving them according to various types of health professionals in different regions.

## Methods

### Design

We conducted an evaluative study based on descriptive qualitative methods [41].

### Context Surrounding the Development of Dashboards

For dashboard development, we used a collaborative approach [42] at the core of learning health system approaches. Such an approach can lead to more relevant, efficient, and sustainable results than a more traditional approach in terms of development and problem-solving in the relevant communities and organizations [43].

In collaboration with the ministerial advisor and the four project managers of the QAP, we selected a format and relevant indicators for this exercise. These choices of format and indicators were then validated with members of the QAP advisory committee, composed of managers, leading clinicians, and anyone involved in the QAP implementation from regional organizations in the province.

A format of a maximum 2-sided sheet was selected (see Multimedia Appendix 1). The choice of a paper rather than digital format was made owing to its simplicity, accessibility (easily accessible to all without depending on technology or connectivity), and instantaneous visibility (without requiring complex navigation). These advantages were prioritized despite the knowledge of disadvantages such as the need for manual updates and space limitations. This support is particularly useful experimentally, even though we acknowledge that it will need to evolve into a digital modality during potential routinization in regular managerial processes.

On the first page, nine indicators were selected from provincial clinical-administrative data from the Quebec Integrated Chronic Disease Surveillance System developed by the Institut national de santé publique du Québec [44]. Indicator selection was based on a conceptual framework on the quality of care for dementia, covering a continuum of care from primary care to emergency use and hospitalizations [45]. Eight of these indicators (type of physician most regularly visited, percentage of people with at least one visit to a family doctor, average number of visits to a family doctor per person, percentage of people with at least one emergency room visit, percentage of people with at least one hospitalization, average number of days hospitalized per person, percentage of people with at least one hospitalization with an alternative care level—representing patients who are hospitalized but no longer requiring acute care and waiting for long-term placement—and average number of days in care level) were measured in 2019-2020 in the population of individuals with dementia 65 years and older, and one indicator (prevalence) was measured between 2000-2001 and 2019-2020 in the population of individuals with dementia 40 years and older. While these surveillance data are highly useful for tracking diseases, they are sometimes underused by local actors due to their excessive quantity and the lack of means for rapid analysis [44]. Data visualization, facilitated by dashboards, makes these data more accessible, facilitating decision-making.

On the second page, we presented findings of a thematic analysis of two sources: annual reports to the Ministry of Health and Social Services produced by the managers involved in the QAP between 2017 and 2019, as well as notes of meetings between the ministerial advisor and the four QAP project managers with the managers involved in the QAP in 2020. While quantitative data highlight high-level trends, qualitative data served to enrich the dashboards by providing context to the figures, offering a more comprehensive and engaging perspective [46].

Finally, we chose to present specific results for the population of each health organization and for the entire province. This choice was made to contextualize the results of each organization to those of the entire province.

### Data Collection

In spring 2022 (April to June), the ministerial advisor and the four QAP project managers (EM, CF, JD, and CM) organized 90-minute virtual meetings with the managers and leading clinicians involved in the QAP implementation as well as other persons involved in the QAP (eg, research personnel) in each of the 24 regional health organizations. These meetings aimed to share the new directions of the QAP and explore the progress and challenges of the pandemic with each organization. The last 30 minutes of these meetings were devoted to presenting regional dashboards and facilitating group interviews by the research team. The research team had approximately 10 minutes to present the dashboards (5 minutes for quantitative aspects and 5 minutes for qualitative aspects) and approximately 20 minutes to conduct a group interview with the participants in the meetings to assess the interest and utility of the dashboards as well as to assess the needs for improvement of these dashboards. The interview guide included four open-ended questions and prompts: “Are these results surprising to you?” “How will this dashboard be useful to you?” “Are there any results missing that you would have wanted in this dashboard?” and “Do you have any questions?”

Four members of the research team alternatively facilitated the group interviews (GAL, MG, YC, and IV). One member (GAL) was present in 20 group interviews, ensuring consistency, reinforcing study fidelity and credibility [47]. Two members were always present: one to present the quantitative part of the dashboards (GAL or IV) and the other to present the qualitative part (MG or YC). In addition, one member was designated the main note-taker, while the second member supported the main note-taker and had to read and complete the notes or suggest modifications on a document shared live and online by the main note-taker.

The note-takers were instructed to note dynamics between the participants and to indicate the participant who put forth each observation noted. Thus, a code was assigned to the interlocutor: manager (ie, director, deputy director, coordinator, unit or service head), leading clinician (ie, territorial nurse, physician, or social worker), other participant (ie, research agent or research coordinator), or an unidentified person (when note-takers had not identified the interlocutor).

Consent was obtained verbally at the onset of meetings, and no audio or video recordings were conducted to streamline the analyses to protect confidentiality, especially given the involvement of project managers in organizing the meetings.

### Ethical Considerations

This study received ethical approval from the ethics committee of the Centre intégré universitaire de santé et de services sociaux de l’Estrie - Centre hospitalier universitaire de Sherbrooke (MP-31-2021-3701) to ensure that all research activities involving human participants adhered to ethical standards and guidelines. The participants provided informed consent before participating in the study, emphasizing their voluntary participation, the purpose of the research, and the confidentiality of their information. All procedures involving human subjects were conducted in accordance with the ethical standards of the ethics committee and the principles outlined in the Declaration of Helsinki. The research team prioritized participant well-being, privacy, and the responsible handling of data throughout the study.

### Analysis

#### Overview

Content analysis was performed on the observation notes [48]. First, a research team member (GAL) read all the notes several times to become familiar with the content. Subsequently, a preliminary version of the coding manual was generated by analyzing the observation notes. Each segment of the notes taken in the observation grid, deemed relevant and related to the research question, was coded and then grouped under conceptual categories. Once the preliminary coding manual was developed, all conceptual categories were defined. A second research team member (MG), who participated in the majority of group interviews, reviewed the manual to ensure its accuracy and consistency. The coding manual was then iteratively revised during the analysis [49] until consensus was reached among all research team members (GAL, MG, YC, and IV).

#### Categorization by Region

Each regional health organization involved in the QAP was categorized into one of three groups using a method used by the Ministry of Health and Social Services [50].

Thus, 13 organizations were categorized in a “university/peripheral regions” group, referring to regions located in a university city where there is a medical school or on the outskirts of such cities. Four organizations were categorized in a “mixed regions” group, where a lack of resources applies to only one part of the territory or where only a part of the territory is considered rural or remote. Seven organizations were categorized in a “remote or isolated regions” group, including organizations not falling into any of the above categories and for which the entire territory is considered remote from urban centers or even isolated. A list of organizations grouped by region is presented in Multimedia Appendix 2.

Similarities and differences were then noted between different types of regions (university/peripheral, mixed, and remote/isolated).

#### Categorization by Type of Participants

As interest, perceived utility, and needs in terms of dashboards could vary among different types of participants (managers, leading clinicians, or other participants), and their degree of participation also varied greatly between group interviews, a specific analysis for each type of participant was conducted.

Similarities and differences were then noted between different types of professionals (managers, leading clinicians, and other participants).

## Results

### Participants

Overall, 23 group interviews were conducted. One organization from a university/peripheral region could not participate due to a significant change in the local governance of the QAP, and two organizations from remote/isolated regions participated in the same group interview. Eighty-two individuals participated in the group interviews: 44 were managers, 32 were leading clinicians, and 7 were other participants (mainly research coordinators).

### Similarities Across Regions

#### Interest

Dashboards received positive reactions from participants in all regions. Some indicators sparked more discussion than others. These discussions focused on whether the results were surprising or not and on participants providing justifications for these results. Participants from all regions were particularly interested in the following indicators: alternative care level, prevalence, emergency room visits, and the type of physician most regularly visited. Participants from all regions were also highly interested in qualitative results, but the varied nature of these results does not allow for a more specific analysis of these discussions (Table 1).

**Table 1:** Codes for the main discussion points related to interest categorized by regions and by type of professionals.^a^

Discussion points		University/peripheral organizations (n=13)					Mixed organizations (n=4)					Remote/isolated organizations (n=7)			
		UI^b^	M^c^	LC^d^	O^e^	UI		M	LC	O	UI		M	LC	O
**Positive reactions**															
	Participants found the results not surprising	2	3	2	0	0		2	0	0	0		3	0	0
	Participants found the results interesting	1	4	3	1	0		2	0	0	0		2	0	0
	Participants did not ask for a copy of the dashboard	0	0	0	0	1		0	0	0	0		0	0	0
	Participant appreciated the format of the dashboard	0	1	0	0	0		0	0	0	0		0	0	0
**Results that were discussed**															
	Only qualitative findings were available	0	0	0	0	0		0	0	0	2		0	0	0
	Prevalence was not available	0	0	0	0	1		0	0	0	0		0	0	0
	Alternate level of care (delayed discharge) indicator	0	4	1	0	0		1	0	0	0		1	0	0
	Prevalence indicator	0	1	1	0	0		1	2	0	0		1	0	0
	Emergency indicators	0	2	1	0	0		1	0	0	0		2	0	0
	Most regularly seen physicians indicator	0	2	1	0	0		0	0	0	0		0	0	0
	Qualitative findings	0	1	0	0	0		1	0	0	0		1	0	0
	References (emergency or hospital)^e^	0	1	1	0	0		0	0	0	0		1	0	0
	Hospitalization indicators	0	1	0	0	0		0	0	0	0		0	0	0

^a^The numbers in each cell represent the number of times the codes were observed during the discussions.

^b^UI: unidentified.

^c^M: managers.

^d^CL: leading clinicians.

^e^O: other.

^e^Despite the fact that no indicators of references were presented, the discussions revolved around references to emergency or hospitalizations.

#### Utility

Participants from all regions offered several reflections on the utility of the dashboards. Primarily, participants believed that their tailored dashboard would be useful for identifying successes and challenges specific to their territory. Moreover, the participants thought the dashboards could mobilize different stakeholders, including the top management of regional health organizations or clinicians from interdisciplinary primary care clinics in their region. In all cases, the reception of dashboards suggested a desire for broader processes of critical thinking, self-examination, and learning on the part of participants (Table 2).

**Table 2:** Codes for the main discussion points related to utility categorized by regions and by type of professionals.^a^

Discussion points			University/peripheral organizations (n=13)									Mixed organizations (n=4)									Remote/isolated organizations (n=7)						
			UI^b^		M^c^		LC^d^		O^e^		UI			M		LC		O		UI			M		LC		O
**Planning or implementation support**																											
	Dashboards could be useful for the local Quebec Alzheimer Plan committees	1		2		2		0		0			2		0		0		0			0		0		0	
	Dashboards could be useful for clinician training	1		1		2		0		0			2		0		0		0			0		0		0	
	Dashboards are useful to identify challenges	1		1		0		0		0			1		0		0		0			1		0		0	
	Dashboards could be useful to follow patients	0		0		0		0		0			1		0		0		0			1		0		0	
	Participants did not react when prompted regarding the dashboard usefulness for the local Quebec Alzheimer Plan committees or training	1		0		0		0		0			0		0		0		0			0		0		0	
**Mobilization**																											
	Mobilize directions	1		3		1		0		0			2		0		0		1			2		0		0	
	Mobilize family physicians	0		0		1		0		0			1		0		0		0			2		0		0	
	Mobilize primary care clinicians (practicing in interdisciplinary groups)	0		1		1		0		0			0		0		0		1			0		0		0	
	Mobilize family physicians (practicing in solo practices or who are uncooperative)	0		0		0		0		0			0		0		0		1			0		0		0	
	Mobilize champions (physicians)	0		1		0		0		0			0		0		0		0			0		0		0	
	Mobilize key actors (no precision on who these key actors are)	0		1		1		0		0			1		0		0		0			0		0		0	
**Other uses**																											
	To modify the perceived role of nurses	0		0		1		0		0			0		0		0		0			0		0		0	
	To know what is done elsewhere	0		1		0		0		0			0		0		0		1			0		0		0	
	Research purposes	0		0		0		1		0			0		0		0		0			0		0		0	

^a^The numbers in each cell represent the number of times the codes were observed during the discussions.

^b^UI: unidentified.

^c^M: managers.

^d^CL: leading clinicians.

^e^O: other.

#### Specific Needs

##### Comparisons

Participants from all regions made proposals or asked clarifying questions suggesting specific needs. The participants appreciated the element of comparisons presented, but many suggested for the dashboard to also present the temporal trend of indicators. Other types of comparisons were also proposed depending on the organizations, without unanimity among regions. For example, participants from a mixed organization would have liked to have a comparison to the metropolis of the province (Montreal), while participants from another mixed organization would prefer to have a comparison to a territory that is geographically or demographically similar or, conversely, to be compared to a territory that is completely different (Table 3).

**Table 3:** Codes for the main discussion points related to needs and questions, categorized by regions and by type of professionals.^a^

Discussion points			University/peripheral organizations (n=13)									Mixed organizations (n=4)									Remote/isolated organizations (n=7)						
			UI^b^		M^c^		LC^d^		O^e^		UI			M		LC		O		UI			M		LC		O
**Comparisons**																											
	Comparisons over time (evolution)	0		1		0		0		0			1		0		0		0			1		0		0	
	Comparisons to the province are useful	0		0		1		0		0			0		0		0		0			1		0		0	
	Comparisons with another territory would be better	0		2		1		0		0			0		0		0		0			0		0		0	
	Comparisons with a specific territory are not important; what matters is to go beyond territorial characteristics	0		1		0		0		0			0		0		0		0			0		0		0	
	Comparison with the province is over too large a scale (no alternative offered)	0		1		0		0		0			0		0		0		0			0		0		0	
	Comparisons with a similar territory in terms of aging population would be better	0		1		0		0		0			0		0		0		0			0		0		0	
	Comparisons to a completely different territory would be interesting	0				0		0		0			0		0		0		0			0		0		0	
Stratifications: stratify by smaller regions within the territory			0		1		0		0		0			1		1		0		0			1		0		0
**Identify specific patients**																											
	Identifying patients registered to an interdisciplinary primary care clinic versus those in other practices would be important	1		1		2		0		0			2		0		0		0			0		0		0	
	Identifying orphan patients would be important	0		3		3		0		0			0		0		0		0			1		0		0	
	Identifying patients who are known to home care services would be important	0		3		1		0		0			1		0		0		0			0		0		0	
	Identifying patients with undiagnosed mental health issues would be important	0		1		0		0		0			0		0		0		0			0		0		0	

^a^The numbers in each cell represent the number of times the codes were observed during the discussions.

^b^UI: unidentified.

^c^M: managers.

^d^CL: leading clinicians.

^e^O: other.

##### Targeted Populations and Other Indicators

Participants from all regions expressed the need to produce results concerning patients not registered in interdisciplinary primary care clinics or without a family doctor (orphan patient), and to produce results on services received by individuals with dementia from health care professionals other than fee-for-service physicians (Table 3). Proposals particularly concerned visits with nurses, social workers, pharmacists, and occupational therapists, without a real consensus on the type of professional (Table 4).

**Table 4:** Codes for the main discussion points related to additional indicators and proposals, categorized by regions and by type of professionals.^a^

Discussion points			University/peripheral organizations (n=13)									Mixed organizations (n=4)									Remote/isolated organizations (n=7)						
			UI^b^		M^c^		LC^d^		O^e^		UI			M		LC		O		UI			M		LC		O
**Other health professional indicators**																											
	Indicators from other professionals would be important; no precision on the type of professionals	0		0		0		0		0			1		0		0		0			2		0		0	
	Indicators from social workers would be important	0		0		2		0		0			0		0		0		0			0		0		0	
	Indicators from occupational therapists would be important	0		0		0		0		0			0		0		0		0			1		0		0	
	Indicators from nurses would be important	0		1		0		0		0			0		0		0		0			0		0		0	
	Indicators from pharmacists would be important	0		1		1		0		0			0		0		0		0			0		0		0	
**Other indicators**																											
	Primary care follow-up indicators (eg, evaluation, support to the care partner) as soon as the diagnosis is made	0		0		1		0		0			0		0		0		0			0		0		0	
	Reasons for the consultation or the hospitalization	0		0		2		0		0			0		1		0		0			0		0		0	
	Home care service use	0		0		0		0		0			1		0		0		0			0		0		0	
	Trajectory indicators	0		1		0		0		0			0		0		0		0			0		0		0	
	Referrals for behavioral and psychological symptoms of dementia	0		1		0		0		0			0		0		0		0			0		0		0	
	Number of beds	0		1		0		0		0			0		0		0		0			0		0		0	
	Number of physicians trained for dementia care	0		0		0		0		0			1		0		0		0			0		0		0	
	Number of human resources (before/after the pandemic)	0		1		0		0		0			0		0		0		0			0		0		0	
	Antidementia medications	0		0		0		0		0			0		0		0		0			1		0		0	
	Satisfaction of patients	0		0		1		0		0			0		0		0		0			0		0		0	
	Timeliness of the diagnosis	0		0		0		0		0			1		0		0		0			0		0		0	
**Other propositions or questions**																											
	Information on how to diagnose, what is a diagnosis allowing	0		0		0		0		0			0		0		0		0			1		0		0	
	Diagnostic tools and training specific for their population (Indigenous)	0		0		0		0		0			0		0		0		0			1		0		0	
	10-year projections of prevalence	0		0		0		0		0			1		0		0		0			0		0		0	
	Dashboards presented monthly	0		1		0		0		0			0		0		0		0			0		0		0	
	More information on how patients were identified	1		1		1		1		0			0		0		0		0			1		0		0	
	Where are the data coming from	0		2		0		0		0			1		0		0		0			0		0		0	

^a^The numbers in each cell represent the number of times the codes were observed during the discussions.

^b^UI: unidentified.

^c^M: managers.

^d^CL: leading clinicians.

^e^O: other.

### Divergences Between Regions

#### Interest

Participants from university/peripheral regions were particularly interested in results on hospitalizations and continuity of care (visits to family physicians). These same results were less emphasized by participants from other regions (mixed or remote/isolated) (Table 1).

#### Utility

Participants from university/peripheral regions were the first to propose that these dashboards could serve as a planning tool for their local QAP steering committees as well as for training clinicians. Following this observation, participants in subsequent focus groups were all invited to directly address this point. Not all responded enthusiastically to this opportunity, and responses varied among participants within the same region, whether peripheral/university, mixed, or remote/isolated. This indicates that perceived utility varies among participants, even among those from university/peripheral regions. Participants from university/peripheral regions also mentioned that these dashboards could be useful for research purposes. This difference likely reflects the level of engagement of regional teams with the QAP (Table 2).

#### Specific Needs

##### Stratifications

Participants from remote/isolated or mixed organizations proposed stratifying data based on smaller territories. They suggested that presenting data for their vast territory did not allow for detecting variations they perceived between different subregions (Table 3).

##### Targeted Populations and Other Indicators

Participants from university/peripheral and mixed regions made several suggestions targeting vulnerable patients or other diverse indicators. For example, they proposed stratifying dashboards or having specific data for patients known in home support or support for elderly autonomy, patients of solo-practicing physicians, and patients with mental health disorders not identified before being diagnosed with dementia (Table 3). Several specific indicators were proposed, but these proposals varied widely between different organizations, whether from university/peripheral or mixed regions. These propositions included: reasons or complexity of hospitalization or consultation, follow-up in interdisciplinary primary care, trajectory indicators, customer satisfaction, home care follow-up, number of clinicians trained under the QAP, timely diagnosis, and projections for the next 10 years (Table 4).

Participants from remote/isolated organizations expressed a need for more information and culturally adapted training for their region. In particular, they expressed the need for diagnostic tools and training adapted to their population, which includes many people from Indigenous communities.

### Similarities and Differences According to Type of Professionals

Leading clinicians who participated in the focus groups mainly came from university/peripheral or mixed organizations. Additionally, university/peripheral organizations were the only ones in which other types of participants (research agents or coordinators) took part in the focus group.

In general, managers and leading clinicians shared the same interest in the prevalence indicator. However, leading clinicians expressed needs that managers and other participants did not express, particularly on follow-up indicators in Family Medicine Groups (interdisciplinary primary care teams), reasons for consultations/visits in primary care or emergencies, and patient satisfaction.

Some managers and research agents or coordinators would have appreciated more information on how individuals with dementia were identified for quantitative indicators. Finally, only one leading clinician and the research agents or coordinators suggested using these dashboards for research purposes.

## Discussion

### Principal Findings

This study determined that dashboards are of interest to those responsible for implementing an Alzheimer Plan in regional health organizations, and their format and content were appreciated regardless of the region or profession of the participants [51]. The study also highlighted specific needs regarding these dashboards that transcend all regions. Participants from all regional health organizations expressed a need for data on orphan patients or those not registered in interdisciplinary primary care clinics, as well as data on indicators related to services offered by other health care and social professionals (ie, nurses, social workers, pharmacists, and occupational therapists). These needs seem to express a desire to better delineate the challenges posed by orphan patients in the organization of health care across the province and to better monitor how interprofessional collaboration takes shape in interdisciplinary primary care clinics (Family Medicine Groups in Quebec).

We also identified interests and specific needs among participants from different regions and different types of professionals. The interest in hospitalization and continuity of care expressed by participants from university/peripheral regions (more urban) can be explained by the higher population density and a diversity of health resources in these regions [31]. Additionally, urban regions often have a greater diversity of health care and social professionals, specialists, and medical technologies available to meet the population’s needs [52]. In contrast, in remote or isolated regions, resources and the number of professionals may be more limited [36,38]. This higher resource availability in urban settings could explain higher interest in continuity of care in urban settings.

Another significant specific need that varied between regions is the need for diagnostic tools and adapted training expressed by participants from remote and isolated regions. The geographical, demographic, and socioeconomic characteristics of these regions make them unique and require different approaches to meet their health needs [1]. Health care professionals in these regions often face different challenges in providing health care for people with dementia [35,36]. This specific need may arise from the observation that both in Quebec and Canada, there is a higher proportion of Indigenous communities in rural areas [53,54], requiring cultural adaptation of care delivery and supporting tools. This is especially important considering the data availability in these regions [55]. Two regions only received qualitative dashboards. Quantitative surveillance data were not available for these organizations either due to their small populations posing risks to data dissemination or due to the fee-for-service mode of physician payment, on which surveillance data rely, but which is less frequent in these regions [56]. The inclusion of qualitative data has proven to be a significant asset in addressing these limitations.

Finally, organizations that span a larger territory (often from remote/isolated or mixed regions) expressed the need for a more granular analysis [42]. Several participants wanted more precise data for smaller territories. However, an ethical constraint prevented us from producing dashboards for smaller territories. Producing a single dashboard for all 24 regional health organizations remains a challenge to explore in future work.

The collaborative approach among researchers, scientific advisors from the National Institute of Public Health of Quebec, QAP project managers, and the QAP ministerial advisor is a major strength of the study [42]. This approach ensured a good understanding of the QAP implementation and formed an alliance between different stakeholders. The research and scientific advisor team could identify the most relevant data, while project managers—as the true points of contact with leading clinicians and managers of different regional health organizations—ensured that the messages were meaningful and well understood by all. The ministerial advisor and the four project managers responsible of the QAP also ensured that the collected and presented data allowed all stakeholders to align with the QAP orientations.

### Limitations

The study also has limitations, particularly in terms of participant acquiescence biases during focus groups. Although the meetings were organized for guidance purposes, managers and leading clinicians from regional health organizations may have felt the need to demonstrate their progress to the ministerial advisor and project managers, as well as to researchers, due to their apparent proximity to the decision-makers and in front of their colleagues. To counter this acquiescence bias, we reiterated the independence of the research team from decision-makers at the beginning of each meeting. Another limitation is the categorization of different regions. Such categorizations are often arbitrary and inconsistent and do not consider the diversity of each region, especially for larger territories, which are often more remote [57]. However, this categorization aligns with that of the Ministry of Health and Social Services and reflects the organization of resources across the different regions of Quebec [50]. Finally, the relatively short time for conducting focus groups (30 minutes) could be considered a limitation. However, in addition to achieving data saturation during focus groups, we conducted these focus groups across all health organizations involved in the QAP and involved several stakeholders (at least one manager was always present, leading clinicians participated in several focus groups, and even research professionals participated in a few), ensuring good representativity of the results.

### Future Work and Recommendations

Considering that the use of dashboards for an Alzheimer Plan is not documented in the scientific literature, future research should focus on the adoption of this tool in different regional health organizations in Quebec and at the governmental level. Studies on the use of dashboards exist for the evaluation of major neurocognitive disorders and the improvement of care offered to these individuals [20-22], but no study specifically mentions the use of a dashboard for an Alzheimer Plan. This will facilitate the transition from an experimental dashboard to a regular tool for managing or monitoring the implementation of an Alzheimer Plan. The composition of focus group participants was different for each region, which may have influenced the results and conclusions. The presence of more leading clinicians in university/peripheral regions may have led to discussions more focused on clinical aspects, while the predominance of managers in mixed or remote/isolated regions may have led to discussions more focused on logistical and organizational challenges related to service delivery in these regions. Other demographic characteristics of participants, such as gender and sex, could have influenced discussions and analyses. It would be interesting for future studies to analyze these differences. Furthermore, no patient or caregiver was part of the focus group, a major element of a learning health system. With increasing incentives to include citizens in health innovations, it will be a great opportunity to study the impact of their perspectives on developing and using dashboards.

### Conclusion

In conclusion, dashboards are part of a learning health system and are a very useful tool for reporting on the challenges and issues related to the implementation of an Alzheimer Plan. However, it is important to consider the differences in the utility and information needs of various regions and types of professionals when developing dashboards to enable an adapted, efficient, and equitable implementation of an Alzheimer Plan that extends to a diverse set of organizations with varied resources. Taking these differences into account in the development of dashboards supporting the implementation of an Alzheimer Plan allows for better meeting the needs of all individuals with major neurocognitive disorders and providing optimal and equitable care, regardless of their region of residence.

## Appendix

Multimedia Appendix 1 Example of an anonymized dashbaord translated in English from French.

Multimedia Appendix 2 Categorization of the regional health organizations.

## Abbreviations

QAP: Quebec Alzheimer Plan (Plan ministériel sur les troubles neurocognitifs majeurs)

## Data Availability

All data generated or analyzed during this study are included in this published article and its appendix files. The comprehensive data set, including anonymized participant information and research materials, is available upon reasonable request. Researchers seeking access to the data for academic, noncommercial purposes can contact the corresponding author to facilitate the sharing of relevant materials. The aim is to promote transparency, reproducibility, and collaboration within the scientific community while respecting the privacy and confidentiality of the participants.

## References

[1] Milieux ruraux et urbains: Quelles différences de santé au Québec?. Institut national de santé publique du Québec.

[2] Foley T, Horwitz L, Zahran R Realising the potential of learning health systems. The Learning Healthcare Project.

[3] Foley T, Boyle S, Jennings A, Smithson WH (2017). "We're certainly not in our comfort zone": a qualitative study of GPs' dementia-care educational needs. BMC Fam Pract.

[4] Enticott J, Johnson A, Teede H (2021). Learning health systems using data to drive healthcare improvement and impact: a systematic review. BMC Health Serv Res.

[5] Le système de santé apprenant ACTE. Alliance for Healthier Communities (Alliance pour des communautés en santé).

[6] Yigitbasioglu OM, Velcu O (2012). A review of dashboards in performance management: implications for design and research. Int J Account Inf Syst.

[7] Ghazisaeidi M, Safdari R, Torabi M, Mirzaee M, Farzi J, Goodini A (2015). Development of performance dashboards in healthcare sector: key practical issues. Acta Inform Med.

[8] Elshehaly M, Randell R, Brehmer M, McVey L, Alvarado N, Gale CP, Ruddle RA (2021). QualDash: Adaptable generation of visualisation dashboards for healthcare quality improvement. IEEE Trans Visual Comput Graphics.

[9] Bhar Layeb S, Omrane Aissaoui N, Hamouda C, Zeghal F, Moujahed H, Zaidi A (2021). Performance indicators and dashboard for an emergency department of a teaching hospital. Tunis Med.

[10] Helminski D, Kurlander JE, Renji AD, Sussman JB, Pfeiffer PN, Conte ML, Gadabu OJ, Kokaly AN, Goldberg R, Ranusch A, Damschroder LJ, Landis-Lewis Z (2022). Dashboards in health care settings: protocol for a scoping review. JMIR Res Protoc.

[11] Lemaire C, Nobre T (2013). Le rôle de la construction d'un tableau de bord dans la trajectoire d'un collectif. Management des Technologies Organisationnelles.

[12] Khairat SS, Dukkipati A, Lauria HA, Bice T, Travers D, Carson SS (2018). The impact of visualization dashboards on quality of care and clinician satisfaction: integrative literature review. JMIR Hum Factors.

[13] Dowding D, Randell R, Gardner P, Fitzpatrick G, Dykes P, Favela J, Hamer S, Whitewood-Moores Z, Hardiker N, Borycki E, Currie L (2015). Dashboards for improving patient care: review of the literature. Int J Med Inform.

[14] Tory M, Bartram L, Fiore-Gartland B, Crisan A, Tory M (2023). Finding their data voice: practices and challenges of dashboard users. IEEE Comput Grap Appl.

[15] Greenhalgh T, Howick J, Maskrey N, Evidence Based Medicine Renaissance Group (2014). Evidence based medicine: a movement in crisis?. BMJ.

[16] Zhuang M, Concannon D, Manley E (2022). A framework for evaluating dashboards in healthcare. IEEE Trans Visual Comput Graphics.

[17] Healthy People 2020 Midcourse Review. Centers for Disease Control and Prevention.

[18] How healthy are people in Canada? An indicators dashboard. Public Health Agency of Canada.

[19] Australia's health performance framework. Australian Institute of Health and Welfare.

[20] Esquer Rochin MA, Gutierrez-Garcia JO, Rosales J, Rodriguez L (2021). Design and evaluation of a dashboard to support the comprehension of the progression of patients with dementia in day centers. Int J Med Inform.

[21] Sharma P, Montgomery RN, Graves RS, Meyer K, Hunt SL, Vidoni ED, Mahnken JD, Swerdlow RH, Burns JM, Mudaranthakam DP (2021). CONSENSUS: a Shiny application of dementia evaluation and reporting for the KU ADC longitudinal Clinical Cohort database. JAMIA Open.

[22] Dalsania P (2006). Dementia dashboard. Top Geriatr Rehabil.

[23] Arsenault-Lapierre G, Henein M, Rojas-Rozo L, Bergman H, Couturier Y, Vedel I (2021). Primary care clinicians' knowledge, attitudes, and practices concerning dementia: they are willing and need support. Can Fam Physician.

[24] Bergman H (2009). Relever le défi de la maladie d'Alzheimer et des maladies apparentée. Une vision centrée sur la personne, l'humanisme et l'excellence. Ministère de la Santé et des Services sociaux du Québec.

[25] Arsenault-Lapierre G, Godard-Sebillotte C, Sourial N, Couturier Y, Bouchard P, Rozo LR, Pilon C, Bergman H, Vedel I (2020). Le Plan Alzheimer québécois, un plan basé sur les soins primaires. Sante Publique.

[26] Guillette M (2021). Plan Alzheimer du Québec. Partager l'expérience de son implantation pour renforcer les soins primaires. Policy brief (format long). Savoirs Université de Sherbrooke.

[27] What is RUISSS?. McGIll University.

[28] Dandy K, Bollman RD (2009). Seniors in rural Canada. Rural and small town Canada analysis bulletin. Agriculture Division, Statistics Canada.

[29] Champagne F, Contandriopoulos AP, Ste-Marie G, Chartran E L'accessibilité aux services de santé et aux services sociaux au Québec. École de santé publique (ESPUM) et Institut de recherche en santé publique Université de Montréal (IRSPUM).

[30] Direction des statistiques sociodémographiques Bulletin sociodémographique Volume 25, numéro 4. Mars 2021. Un aperçu de la situation démographique au Québec en 2020. Statistiques Quebec.

[31] (2009). Entre adaptabilité et fragilité: les conditions d'accès aux services de santé des communautés rurales et éloignées. Institut national de santé publique du Québec.

[32] Guillette M, Couturier Y, Moreau O, Gagnon D, Bergman H, Vedel I (2019). Gouvernance et accompagnement du changement: le cas de la phase expérimentale du Plan Alzheimer du Québec. Innovations.

[33] Régions sociosanitaires du Québec. Ministère de la Santé et des Services sociaux.

[34] Constantinescu A, Li H, Yu J, Hoggard C, Holroyd-Leduc J (2018). Exploring rural family physicians’ challenges in providing dementia care: a qualitative study. Can J Aging.

[35] Giebel C, Hollinghurst J, Akbari A, Schnier C, Wilkinson T, North L, Gabbay M, Rodgers S (2021). Socio-economic predictors of time to care home admission in people living with dementia in Wales: a routine data linkage study. Int J Geriatr Psychiatry.

[36] Innes A, Morgan D, Kosteniuk J (2011). Dementia care in rural and remote settings: a systematic review of informal/family caregiving. Maturitas.

[37] (2021). Mandat sur les performances des soins et services aux aînés- COVID-19. Portrait: les milieux de vie pour aînés au Québec.Portrait: les milieux de vie pour aînés au Québec. Gouvernement du Québec. Commissaire à la santé et au bien être.

[38] Morgan DG, Crossley M, Kirk A, D'Arcy C, Stewart N, Biem J, Forbes D, Harder S, Basran J, Dal Bello-Haas V, McBain L (2009). Improving access to dementia care: development and evaluation of a rural and remote memory clinic. Aging Ment Health.

[39] Mori K, Christodoulou A (2012). Review of sustainability indices and indicators: towards a new City Sustainability Index (CSI). Environ Impact Assess Rev.

[40] Scipioni A, Mazzi A, Mason M, Manzardo A (2009). The Dashboard of Sustainability to measure the local urban sustainable development: the case study of Padua Municipality. Ecol Indicat.

[41] Depover C, Karsenti T, Komis V, Karsenti T, Savoie-Zajc L (2011). La recherche en éducation: étapes en approches. La Recherche Évaluative.

[42] Morrissette J (2013). Recherche-action et recherche collaborative. NPS.

[43] Foley T, Vale L (2023). A framework for understanding, designing, developing and evaluating learning health systems. Learn Health Syst.

[44] Concannon D, Herbst K, Manley E (2019). Developing a data dashboard framework for population health surveillance: widening access to clinical trial findings. JMIR Form Res.

[45] Sourial N, Godard-Sebillotte C, Bronskill SE, Hacker G, Vedel I (2022). Quality indicator framework for primary care of patients with dementia. Can Fam Physician.

[46] Baysal O, Holmes R, Godfrey MW (2013). Developer dashboards: the need for qualitative analytics. IEEE Softw.

[47] Drapeau M (2004). Les critères de scientificité en recherche qualitative. Pratiques Psychol.

[48] Lefebvre B (1989). La recherche qualitative et l'analyse de contenu en éducation. Can J Educ.

[49] Schreier M, Atkinson P, Delamont S, Cernat A, Sakshaug JW, Williams RA (2019). Content analysis, qualitative. Social Media Analysis.

[50] Les régions SARROS. SARROS.

[51] Strumpf E, Goldsmith LJ, King CE, Lavergne R, Mccracken RK, McGrail KM, Simon L (2022). Mesure l'accès et la qualité des soins de première ligne au Québec: Réflexions issues de recherches sur la prise en charge des patients. CIRANO.

[52] Répartition des médecins selon la région administrative. Collège des Médecins de Québec.

[53] Population growth in Canada's rural areas, 2016 to 2021. Statistics Canada.

[54] (2023). Chapter 2. Profile of Indigenous Canada: Trends and data needs. OECD Library.

[55] Backonja U, Park S, Kurre A, Yudelman H, Heindel S, Schultz M, Whitman G, Turner AM, Marchak NT, Bekemeier B (2022). Supporting rural public health practice to address local-level social determinants of health across Northwest states: Development of an interactive visualization dashboard. J Biomed Inform.

[56] Gagnon R, Rochette L, Plante C (2017). Cadre de qualité des données du Système intégré de surveillance des maladies chroniques du Québec. Institut national de santé publique du Québec.

[57] Danek R, Blackburn J, Greene M, Mazurenko O, Menachemi N (2022). Measuring rurality in health services research: a scoping review. BMC Health Serv Res.

