# Supplementary material for: Dashboards to Support Implementation of the Quebec Alzheimer Plan: Evaluation Study With Regional and Professional Considerations
Source: JMIR Form Res. 2024 May 8;8:e55064. doi: 10.2196/55064 (PMC11112472; doi:10.2196/55064)
Supplement: Multimedia Appendix 1 [file formative_v8i1e55064_app1.pdf]

Persons with dementia:
What happened in Your Organization and in the province?

The number of persons diagnosed with dementia is increasing rapidly. As a result, Quebec has developed an Alzheimer Plan to prepare the healthcare system to better manage persons with dementia. Here is how your organization compares to Quebec as a whole in 2019-2020 with regard to persons with dementia.

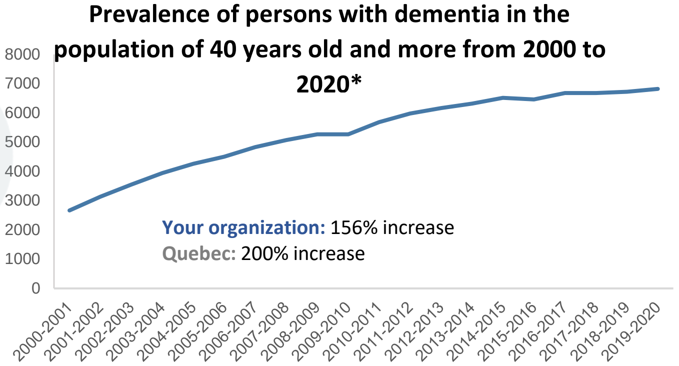

Which doctor do persons with dementia in your organisation see the most? And how often?

Family physicians are the doctors most often in contact with persons with dementia. Our research has also shown that family physicians participating in the Quebec Alzheimer Plan have good attitudes, knowledge, and practices, and that they improve the quality of care for persons with dementia.

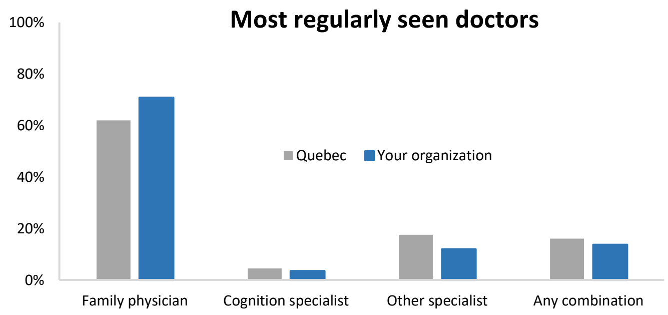

Percentage of persons with at least one visit to the family physician and mean number of visit per person

Quebec
79,4 %
3,4 visits

Your organization
78,7 %
3,6 visits

Which acute care services the persons with dementia in your organization use?

Our research has shown that persons with dementia use hospital services 2 times more than elderly persons without dementia. High hospital use can be reduced by better upstream care. Indeed, our research has shown that if these persons with dementia see their family doctor regularly, they are less likely to go to the emergency department and are less likely to be hospitalized.

Percentage of persons with dementia with at least one emergency department visit*

Quebec
49,3 %

URGENCES

Your organization
48,6%

Percentage of persons with at least one hospitalization and mean number of days hospitalized[T] **

Quebec
29,1 %
20,1 days

Your organization
28,9%
24,1 days

Percentage of persons with at least one hospitalisation with delayed discharge (alternate level of care)[ŧ] and mean number of days

Quebec
4,8 %
46,3 days

NSA

Your organization
3,8 %
40,9 days

Notes : * Indicators available via Infocentre. Dementia is underestimated and represent only persons identified via administrative databases. **Includes long-term hospitalisations and psychiatric hospitalisations but excludes hospitalisations with alternate level of care. Ŧ Alternate level of care is when a patient is still hospitalized but no longer requires acute care services, often waiting for long-term care admission.

McGill University
Université de Sherbrooke
ROSA Recherche en organisation des services sur l'Alzheimer
Institut national de santé publique Québec

Implantation of the Quebec Alzheimer Plan:
What is going on in your organisation and across the province?

Success stories in Quebec and facilitators for the implementation of the Plan

- ✓ The quality of change management.
- ✓ The increase in the capacity and skills of family medicine team nurses.
- ✓ The emergence of a sense of confidence in interprofessional teamwork in family medicine teams.
- ✓ A perceived increase in the number of cognitive assessments and diagnoses carried out in family medicine teams.
- ✓ Better coordination between primary care and secondary care.

Challenges in Quebec and barriers to implementing the Plan

- × Transfer of leadership from family medicine teams to regional health organizations
- × Weak continuity between family medicine teams and directions for Support Program for the Autonomy of Seniors
- × The role of Social Workers in family medicine teams is poorly explained and asserted.
- × Getting some physicians on board remains a challenge.
- × Follow-up of persons with dementia is often poorly implemented.
- × Inability of regional health organizations and Ministry of Health and Social Services to measure changes in family medicine teams practice.
- × COVID pandemic and post-pandemic relaunch.

Success stories in your organization

- ✓ Clear project governance, proactive steering committee meetings and promotion of change at territorial tables.
- ✓ A very active and rigorous steering committee meets regularly to identify issues and make adjustments (prior to pandemic).
- ✓ Pharmacists, social workers, and community organizations are integrated into the interdisciplinary primary care process.

Challenges in your organization

× Difficulty identifying family medicine group pharmacists and insufficient number of hours worked in family medicine group.

× Family medicine group nurses do not systematically refer patients to family medicine group's pharmacists for a complete medication review.

× A concern for the follow-up of intermediate resource users: difficulty in getting the intermediate resource promoter's staff to apply the Ministerial Plan's recommendations, and high staff turnover.

This dashbaord of was created by Dr. Isabelle Vedel, Dr. Geneviève Arsenault-Lapierre, Dr. Yves Couturier and M. Maxime Guillette of the Research in Organization of services for Alzheimer's disease (ROSA) team and Ms. Victoria Massamba of the Institut national de santé publique (INSPQ). Acknowledgements to Dr. Claire Godard-Sebillotte, Ms. Mary Henein and Ms. Juliette Champoux-Pellegrin of the ROSA team, M. Louis Rochette, M. Éric Pelletier and Ms. Sylvie Muller of the INSPQ, and to the four project managers of the Quebec Alzheimer Plan.

McGill University · Université de Sherbrooke · ROSA Recherche en organisation des services sur l'Alzheimer · Institut national de santé publique Québec
