# Supplementary material for: Dashboards to Support Implementation of the Quebec Alzheimer Plan: Evaluation Study With Regional and Professional Considerations
Source: JMIR Form Res. 2024 May 8;8:e55064. doi: 10.2196/55064 (PMC11112472; doi:10.2196/55064)
Supplement: Multimedia Appendix 2 [file formative_v8i1e55064_app2.docx]

**Appendix 2 – Categorization by region**

**University/Peripheral Regions**

This category includes a total of 13 regional health organizations: the five Integrated University Health and Social Services Centers (CIUSSS) in Montreal (CIUSSS de l'Ouest-de-l'Île-de-Montréal, CIUSSS du Centre-Ouest-de-l'Île-de-Montréal, CIUSSS du Centre-Sud-de-l'Île-de-Montréal, CIUSSS du Nord-de-l'Île-de-Montréal, and CIUSSS de l'Est-de-l'Île-de-Montréal), the CIUSSS de la Capitale-Nationale, CIUSSS de l'Estrie – Centre hospitalier universitaire de Sherbrooke, the Integrated Health and Social Services Center (CISSS) de Laval, the CISSS de Lanaudière, the three CISSS of Montérégie (CISSS de la Montérégie-Est, CISSS de la Montérégie-Ouest, CISSS de la Montérégie-Centre), and the CISSS de Chaudière-Appalaches.

**Mixed Regions**

This category includes four regional health organizations: the CISSS du Saguenay-Lac-Saint-Jean, the CISSS de la Mauricie-et-Centre-du-Québec, the CISSS de l'Outaouais, and the CISSS des Laurentides.

**Remote/Isolated Regions**

This category includes seven regional health organizations: the CISSS du Nord-du-Québec, the CISSS de la Gaspésie, the CISSS des Îles-de-la-Madeleine, the Cree Board of Health and Social Services of the James Bay, the regional health and social services center of Baie-James, the CISSS de l'Abitibi-Témiscamingue, and the CISSS du Bas-Saint-Laurent.
